# Homotaurine Induces Measurable Changes of Short Latency Afferent Inhibition in a Group of Mild Cognitive Impairment Individuals

**DOI:** 10.3389/fnagi.2014.00254

**Published:** 2014-09-23

**Authors:** Alessandro Martorana, Francesco Di Lorenzo, Guglielmo Manenti, Roberta Semprini, Giacomo Koch

**Affiliations:** ^1^System Medicine Department, Clinica Neurologica-Memory Clinic, Università di Roma “Tor Vergata”, Rome, Italy; ^2^Non-Invasive Brain Stimulation Unit, Department of Behavioral and Clinical Neurology, Santa Lucia Foundation IRCCS, Rome, Italy; ^3^Department of Diagnostic Imaging and Interventional Radiology, Molecular Imaging and Radiotherapy, Fondazione Policlinico “Tor Vergata”, Rome, Italy; ^4^ACISMOM Centro Diabetologico, Rome, Italy

**Keywords:** MCI, SLAI, homotaurine, GABA, cortical interneurons

## Abstract

Current treatment options for patients with Alzheimer’s disease (AD) are limited at providing symptomatic relief, with no effects on the underlying pathophysiology. Recently, advances in the understanding of the AD pathogenesis highlighted the role of ABeta (Aβ) oligomers particularly interfering with mechanisms of cortical plasticity such as long-term potentiation (LTP) and long-term depression (LTD). These findings led to the development of potential anti-amyloid therapies, and among them homotaurine, a glycosaminoglycan mimetic designed to interfere with the actions of Aβ early in the cascade of amyloidogenic events, and by its γ-aminobutyric acid type (GABA) A receptor affinity. Recently, we showed that AD patients have impaired LTP-like cortical plasticity, as measured by standard theta burst stimulation protocols applied over the primary motor cortex (M1). Furthermore, AD patients have a weakened short latency afferent inhibition (SLAI), a neurophysiological measure of central cholinergic transmission, which changes reflect the cholinergic dysfunction occurring in the pathology. Here, we aimed at investigating whether homotaurine administration could modulate *in vivo* measured mechanisms of synaptic plasticity, namely LTP and LTD, and also SLAI in a group of mild cognitive impaired patients. We observed that homotaurine administration did not induce relevant changes of both LTP and LTD recordings, while induced changes of SLAI in our group of patients. We suggest that homotaurine effects are dependent on changes of cortical GABA transmission suggesting a potential role for this compound in ameliorating the cholinergic transmission by modulating the inhibitory cortical activity.

## Introduction

Mild cognitive impairment (MCI) is a cognitive status difficult to define. It represents the gray area set between the normal and pathologic cognition. Its definition and description of clinic-pathological features depends on the different methods used to assess MCI (Petersen et al., 2014). Given its dynamic evolution, it is in general considered a pre-clinical condition during which pathological changes may occur long before cognitive decline appear. MCI could evolve in cognitive decline of Alzheimer’s type, fronto-temporal dementia, Parkinson’s disease, etc. (de Mendonça et al., 2004, 2005; Aarsland et al., 2011), and in this view, features like biomarkers, neuropsychological assessment, and radiological signs could be helpful to define its nature. MCI of Alzheimer’s dementia type is suggested to recognize the same pathological background, although factors responsible for its evolution toward AD remain unknown (Grundman et al., 2004). AD is an age-related neurodegenerative disorder responsible for progressive cognitive decline and dementia. It is the most diffuse form of dementia worldwide. Pathologic hallmark of AD is extracellular senile plaques and intracellular neurofibrillary tangles. The pathogenesis of AD is debated. Current and leading pathogenic hypothesis is the so-called amyloid hypothesis (Hardy and Selkoe, 2002). The amyloid precursor protein (APP) is a transmembrane protein with unknown function. Changes in APP proteolytic processing are considered central in AD pathogenesis, where an imbalance between amyloidogenic (beta and gamma-secretase mediated) and non-amyloidogenic (alpha-secretase mediated) pathways, could be responsible for the overproduction of insoluble amyloid peptides. The progressive accumulation of these peptides, associated with a reduced clearance, would promote aggregation of Aβ peptides in oligomers and fibrils, species with a powerful toxic effect on neurons responsible for dramatic changes of synaptic plasticity machinery with impaired long-term potentiation (LTP) and prolonged long-term depression (LTD), spine shrinkage and loss, and neuronal degeneration (Shankar et al., 2008; Palop and Mucke, 2010). These changes are supposed to start long before cognitive decline appear, therefore, low Aβ levels could be easily detected in MCI patients. Since that, MCI is often considered a state during which therapies to delay or modify pathologic cascade of AD could be taken (Simon et al., 2012; Ghosh et al., 2013). Homotaurine or Tramiprosate, is a small aminosulfonate compound naturally found in seaweeds. It is a compound with features typical of glycosaminoglycanes (GAGs) (Wright, 2006). These are compounds able to induce and enhance Aβ aggregation, fibrillogenesis with neurotoxic effect on neurons. Homotaurine on the contrary has been shown to be able to counteract Aβ fibrillogenesis reducing the toxic effects on neurons. Indeed, in experimental models homotaurine administration induced inhibition of Aβ fibrils formation, reduction of the brain pathologic burden, and formation of physiological LTP (Gervais et al., 2007). Moreover, early studies made in humans showed that homotaurine induces a dose-dependent reduction of Aβ1-42 cerebrospinal levels in AD patients (Aisen et al., 2007), with reduction of brain atrophy (Gauthier et al., 2009) and positive cognitive measurable effects (Saumier et al., 2009). These features suggest potential neuroprotective effects of homotaurine that could be useful in therapeutic strategies in early phases of AD (Caltagirone et al., 2012). Besides the anti-amyloid action, homotaurine has direct effects also on neuronal activity as a modulator of excitatory neurotransmission, due to its binding affinity for GABAA receptors (GABAARs). This modulatory action strengthens the potential neuroprotection mechanisms of homotaurine. Such potential neuroprotective effects could be useful for MCI patients to delay progression to cognitive decline, although few literature addressed such target.

Here, we aimed to verify whether homotaurine administration could induce changes of cortical activity *in vivo*, measurable with electrophysiological tools validated for humans. The aim of this work was to study the effects of homotaurine on motor cortical excitability using standard paired-pulse protocols assessing short intracortical inhibition (SICI) and intracortical facilitation (ICF) and on cholinergic transmission using short latency afferent inhibition (SLAI) paradigm, in a group of MCI patients. Each patient was evaluated for plasticity induction of LTP/LTD-like effects using respectively intermittent TBS (iTBS) or continuous TBS (cTBS) protocols of repetitive transcranial stimulation as well. Parallel neuropsychological assessment was performed at the baseline and following 4 weeks treatment.

## Materials and Methods

### Subjects

We examined 10 patients with a diagnosis of amnestic MCI according to the Petersen criteria. The mean (±SD) age of the patients was 61.9 (±1.9) years (for patients characteristics see Table 1). All patients underwent a complete clinical investigation, including medical history, neurological examination, mini mental state examination (MMSE), a complete blood screening (including routine exams, thyroid hormones, level of B_12_), neuropsychological examination (Pierantozzi et al., 2004), a complete neuropsychiatric evaluation, and neuroimaging consisting of magnetic resonance imaging (1.5 T MRI). Exclusion criteria were the following: (1) patients with isolated deficits and/or unmodified MMSE (≥25/30) on revisit (6, 12, and 18 months follow-up), patients with clinically manifest acute stroke in the last 6 months showing an Hachinsky scale >4, and a radiological evidence of sub-cortical lesions. None of patients revealed pyramidal and/or extrapyramidal signs at the neurological examination. At the time of enrollment, in the 30 days before participating in this study, none of the patients had been treated with drugs that might have modulated cerebral cortex excitability such as antidepressants, or any other neuroactive drugs (i.e., benzodiazepines, anti-epileptic drugs, or neuroleptics), and they had not been treated with cholinesterase inhibitors. The study was performed according to the Declaration of Helsinki and approved by the ethics committee of the Tor Vergata University in Rome. The Local Ethic Committee approved the study procedures.

**Table 1:** **Demographic characteristic of the MCI patients**.

	Gender	Education	Age
1	F	5	72
2	F	13	72
3	M	2	59
4	F	5	70
5	F	3	74
6	M	3	73
7	M	8	70
8	M	5	73
9	M	13	64
10	M	8	63

#### Drug administration

Homotaurine 100 mg was administered for a period of 4 weeks in order to maximize the pharmacological effect of the drug. All participants were tested on Monday before and after the 4 weeks of homotaurine administration. To guarantee the reliability of TMS results, physicians involved in recordings were completely blinded to the kind of experiments used in this protocol. None of the patients receiving homotaurine complained for side effects.

#### TMS procedure

Single and paired TMS of the motor cortex of both hemispheres were performed with a 9 cm figure-of-eight coil connected with one or two Magstim 200 stimulators (The Magstim Company, Whitland, UK) via one Bistim module. The magnetic stimuli had a nearly monophasic pulse configuration, with a rise time of 100 μs, decaying back to zero over 0.8 ms. For paired-pulse protocols, the output of each of the two Magstim 200 stimulators was connected to the TMS coil using a cable. The coil was placed at the optimal position for eliciting motor-evoked potentials (MEPs) from the contralateral first dorsal interosseous (FDI) muscle. The optimal position was marked on the scalp with a felt pen to ensure the identical placement of the coil throughout the experiment. The handle of the coil pointed backward and was perpendicular to the presumed direction of the central sulcus, about 45° to the midsagittal line. The direction of the induced current was from posterior to anterior, and was optimal to activate the motor cortex transsynaptically (Werhahn et al., 1994). The resting motor threshold (RMT) was defined as the lowest intensity that produced MEPs of >50 μV in at least 5 of 10 trials with the muscles relaxed (Rossini et al., 1994). The active motor threshold (AMT) was defined as the lowest intensity that produced MEPs of >200 μV in at least 5 of 10 trials when the subject made a 10% of maximum contraction using visual feedback (Rothwell, 1997). Determination of RMT and AMT was done in step width of 1% of MSO. SICI and ICF were tested using paired TMS with a subthreshold conditioning stimulus (CS) preceding a suprathreshold test stimulus (TS) (Kujirai et al., 1993; Rothwell, 1997). Subthreshold CS stimulus was set at 80% AMT while the intensity of TS was adjusted to evoke a MEP of 1 mV peak-to-peak in the relaxed left FDI. Interstimulus intervals (ISIs) of 1, 2, 3, 5, 7, 10, and 15 ms were utilized to test SICI and ICF.

#### Short latency afferent inhibition protocol

Short latency afferent inhibition was studied using the technique that has been recently described (Sailer et al., 2003; Lang et al., 2008). CS were single pulses (200 μs) of electrical stimulation applied through bipolar electrodes to the right median nerve at the wrist (cathode proximal). The intensity of the CS was set at just over motor threshold for evoking a visible twitch of the thenar muscles. The intensity of the test cortical magnetic stimulus was adjusted to evoke a muscle response in relaxed right FDI with an amplitude of 1 mV peak-to-peak. The CS to the peripheral nerve preceded the magnetic TS by different ISIs. ISIs were determined relative to the latency of the N20 component of the somatosensory evoked potential induced by stimulation of the right median nerve. The active electrode for recording the N20 potential was attached 3 cm posterior to C3 (10–20 system) and the reference was 3 cm posterior to C4. Five hundred responses were averaged to identify the latency of the N20 peak. ISIs from the latency of the N20 minus 4 ms to the latency of the N20 plus 8 ms were investigated in steps of 4 ms. Ten stimuli were delivered at each ISI. The subject was given audiovisual feedback at high gain to assist in maintaining complete relaxation. The inter-trial interval was set at 5 s (±10%), for a total duration of 5 min. Measurements were made on each individual trial. The mean peak-to-peak amplitude of the conditioned MEP at each ISI was expressed as a percentage of the mean peak-to-peak amplitude size of the unconditioned test pulse in that block.

#### Theta burst stimulation protocol

Motor-evoked potentials were recorded from the right FDI muscles using 9 mm diameter, Ag–AgCl surface cup electrodes. Responses were amplified with a Digitimer D360 amplifier (Digitimer Ltd., UK) through filters set at 20 and 2 kHz, then recorded by computer using SIGNAL software, at a sampling rate of 5 kHz per channel (Cambridge Electronic Devices, UK). A monophasic Magstim 200 device (Magstim Co., UK) was used to define the motor hot-spot and to assess MEP size. The motor hot-spot was defined as the location where TMS consistently produced the largest MEP size at 120% of RMT in the target muscle (Rossini et al., 1994). A second coil was connected to a biphasic Super Rapid Magstim stimulator (Magstim Co., UK) to deliver rTMS. In the theta burst stimulation (TBS) protocols bursts were repeated at 5 Hz (i.e., every 200 ms), while each burst consisted of three stimuli repeating at 50 Hz. For iTBS, a 2 s train of TBS at 80% AMT was repeated 20 times, every 10 s for a total of 190 s (600 pulses) (Huang et al., 2005). Twenty MEPs were collected and averaged at baseline. The intensity of the test pulse was adjusted throughout the experiment so that it evoked a MEP of about 1 mV peak-to-peak amplitude in each individual. Then, over the same hot-spot, 20 MEPs were recorded at 1–5, 6–10, 11–15, 16–20, and 21–25 min after rTMS, and averaged. SLAI was investigated using the technique that has been recently described (Sailer et al., 2003; Lang et al., 2008), where the CS is a single pulse (200 μs) of electrical stimulation applied through bipolar electrodes to the right median nerve at the wrist (cathode proximal). The intensity of the CS was set at just over motor threshold for evoking a visible twitch of the thenar muscles. The intensity of the cortical magnetic TS was adjusted to evoke a muscle response in the relaxed right FDI with an amplitude of about 1 mV peak-to-peak. The CS to the peripheral nerve preceded the cortical magnetic TS by different ISIs, which ranged from −4 to +8 ms from the N20 component, in steps of 4 ms (Martorana et al., 2013). Ten stimuli were delivered at each ISI. Subjects were provided with audiovisual feedback at high gain, in order to assist them in maintaining complete relaxation. The inter-trial interval was set at 5 s (±10%), for a total duration of 5 min. Measurements were made on each individual trial. The mean peak-to-peak amplitude of the conditioned MEP at each ISI was expressed as a percentage of the mean peak-to-peak amplitude of the unconditioned test pulse in that block.

#### Cognitive assessment

All the patients underwent a neuropsychological examination including a general cognitive index (Mini Mental Examination State (Martorana et al., 2013) and the following neuropsychological tests for the specific cognitive domains: (1) 15 Rey’s Word List (Immediate and 15-min Delayed recall) to investigate verbal episodic long-term memory (Magni et al., 1996); (2) forward and backward Digit span (Carlesimo et al., 1996) and the forward and backward Corsi Block Tapping task (Orsini et al., 1987) to investigate short-term memory and working memory; Frontal Assessment Battery (FAB) to investigate executive functions (Appollonio et al., 2005). Parallel versions were used when available.

### Data analysis

Data were analyzed using SPSS for Windows version 11.0; we measured the percentage of change of peak-to-peak amplitudes of the mean MEPs at baseline for each subject in each condition. At baseline two-way repeated measure ANOVAs were performed for each protocol (cTBS and iTBS) with TIME (1–5, 6–10, 11–15,16–20, and 21–25 min after TBS) as *within subjects* factor. To test the effects of the drugs, different repeated measure ANOVAs were performed for each protocol (cTBS and iTBS) with SESSION (baseline vs. end of treatment) and TIME (1–5, 6–10, 11–15, 16–20, and 21–25 min after TBS) as *within subjects* factors.

For SLAI, the electrophysiological parameters of AD patients were analyzed by means of repeated measures ANOVA with ISI 16, 20, 24, and 28 ms(−4, 0, +4, and +8 ms plus the latency of the N20) and TIME (before vs. after homotaurine administration) as within subjects factors.

For SICI and ICF, we performed a repeated measures analysis ANOVA with ISI (1, 2, 3, 5, 7, 10, and 15 ms) and TIME (before vs. after homotaurine administration) as within subjects factors.

The Greenhouse–Geisser correction was used for non-spherical data. When a significant main effect was reached, paired *t*-tests with Bonferroni correction (*p* < 0.05) were employed to characterize the different effects for the specific ISIs. Mauchley’s test examined for sphericity. For all statistical analyses, a *p* value <0.05 was considered to be significant.

## Results

The entire procedure was well tolerated in all subjects. The mean (standard deviation) AMT (calculated as a percentage of maximal stimulator output, MSO) for TMS was PLC: 35.2%.

**Figure 1:**
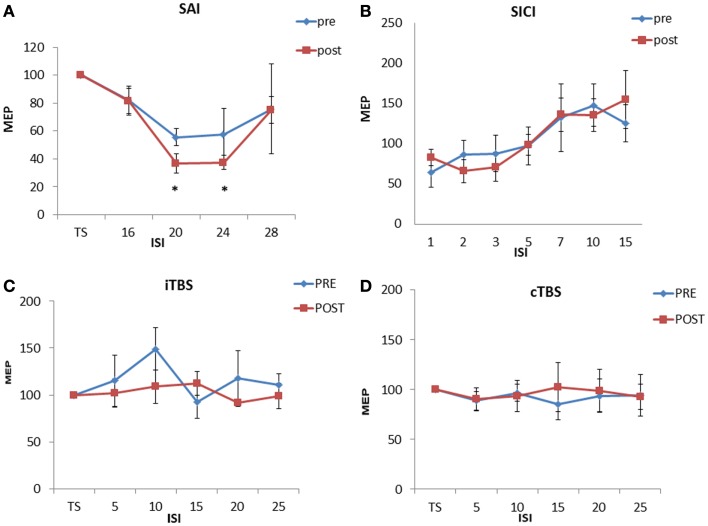
**Effects of 4 weeks of homotaurine-based treatment on neurophysiological parameters**. Homotaurine increased SAI efficiency **(A)**. No effect was observed for SICI and ICF **(B)**, iTBS **(C)**, and cTBS **(D)** protocols.

Repeated ANOVA analysis performed on SLAI measurements revealed a significant ISI [*F*(3,27) = 12.68, *p* = 0.00002] main factor; the interaction ISI × TIME was significant [*F*(3, 27) = 3.65, *p* = 0.024]. *Post hoc* analyses showed that homotaurine increased SAI at ISIs 20 ms (*p* = 0.047) and 24 ms (*p* = 0.031) (Figure 1A). The ANOVA analysis performed on SICI and ICF measurements revealed a significant ISI [*F*(6, 30) = 5.88, *p* = 0.00038] main factor; the interaction ISI × TIME was not significant [*F*(6, 30) = 0.51, *p* = 0.79] (Figure 1B). Homotaurine did not induce any effect on the iTBS (Figure 1C) protocol as shown by the ANOVA [SESSION main factor: *F*(1, 9) = 1.08, *p* = 0.32; TIME main factor: *F*(4, 36) = 1.63, *p* = 0.19; interaction SESSION × TIME: *F*(8, 1) = 0.70; *p* = 0.78]. Finally, Homotaurine did not induce any effect on the cTBS protocol as shown by the ANOVA [SESSION main factor: *F*(1, 9) = 0.07, *p* = 0.78; TIME main factor: *F*(4, 36) = 0.17, *p* = 0.95; interaction SESSION × TIME: *F*(4,36) = 0.40; *p* = 0.81] (Figure 1D).

Neuropsychological scores obtained at baseline and with those obtained after 4 weeks of treatment were compared (see also Table 2). MMSE, RAVLT, Digit span, Corsi span, and FAB scores did not vary after 4 weeks of treatment with homotaurine.

**Table 2:** **Neuropsychological assessment of MCI patients**.

Pre									Post							
	MMSE	FAB	Rey R. I.	Rey R. D.	VS FW	SS FW	VS BW	SS BW	MMSE	FAB	Rey R. I.	Rey R. D.	VS FW	SS FW	VS BW	SS BW
1	26.3	13.5	29.74	5.66	5.15	4.58	3	4	26.3	14.5	27.74	6.66	4.15	4.58	4	3
2	25.7	14.3	23.93	0	4.44	4.18	4	4	25.7	16.3	29.93	3.47	5.44	4.18	4	4
3	25	16.3	36.94	6.8	4.71	4.34	4	2	25	14.3	40.94	8.8	4.71	4.34	2	3
4	25.7	14.3	28.95	6.39	5.17	3.55	3	3	25.3	12.3	39.95	9.39	6.17	3.55	3	3
**5**	26.7	14	25.32	4.37	4.39	5.72	2	4	25.3	14	25.32	4.37	4.39	5.72	2	4
6	26.7	12	26.91	4.23	4.4	4.52	3	5	26.7	14	29.91	0	4.4	5.52	4	5
7	29	8.3	21.34	0	3.55	3.64	3	3	29	8.3	21.34	0	3.55	3.64	3	3
8	28.5	13.2	27.59	3.9	4.53	4.36	3.14	3	29	13.2	27.59	3.9	4.5	4.3	3.1	4
9	28	7.5	21.34	0	5.16	4.56	5	3	28.3	7.5	21.34	0	5.16	4.56	5	3
10	27	12.6	26.89	3.4	4.61	4.38	3.34	3	27	12.6	26.8	3.4	4.6	4.3	3.3	3

*For each administered test appropriate adjustments for sex, age, and education were applied according to the Italian normative data. Neuropsychological measurements did not differ after 4 weeks of treatment with homotaurine. MMSE, mini mental state examination; FAB, frontal assessment battery; REY R. I., Rey recall immediate; Rey R. D., Rey recall delayed; VS FW, verbal span forward; SS FW, spatial span forward; VS BW, verbal span backward; SS BW, spatial span backward*.

## Discussion

The anti-amyloid properties associated to the GABA profile, make homotaurine a new potential drug for treatment of AD (Caltagirone et al., 2012). Since most of the pathological changes observed in AD have been shown to start long before cognitive decline appears (Mattsson et al., 2009; Lim et al., 2014), an appropriate treatment strategy would indicate homotaurine for patients with MCI, a condition that potentially reverse to AD. Homotaurine has been introduced in pharmacotherapy only recently and literature on MCI is scarce. Here, we tested the effects of homotaurine on excitability of the motor cortex and on synaptic plasticity mechanisms by means of neurophysiological protocols on individuals with MCI. Following 4 weeks of treatment, homotaurine induced changes of SLAI cortical inhibitory circuit, without changes of SICI, but was unable to induce changes of the LTP/LTD mechanisms. It is questionable whether the effects we measured are the consequence of anti-amyloid activity or of the high affinity binding of homotaurine for GABAARs. We suppose that the latter is the most likely effect. Cortical neurones are excitatory glutamatergic neurones that project downstream to spinal cord and in part to basal ganglia system. Pyramidal neurons excitability is modulated by the inhibitory activity of the intracortical GABA interneurons. It is supposed that GABA released from interneurons would act on GABAAR expressed on pyramidal neurones. Such inhibitory effect could be reliably measured by paired TMS protocol. The measure obtained called SICI is the result of the GABA intracortical interneurons activation. SLAI instead is a neurophysiological tool that in general is considered a measure of inhibitory interactions between afferent input and motor output. It is supposed that cholinergic system modulates these inhibitory interactions, and that GABA-PV interneurons drive these interactions. SLAI measures are altered in patients suffering from cognitive decline of Alzheimer’s type (Di Lazzaro et al., 2002), and are reported to be normal in MCI patients (Sakuma et al., 2007). SICI and SLAI are two different forms of cortical inhibition (Ziemann et al., 1996; Di Lazzaro et al., 1998; Tokimura et al., 2000). Both can be segregated by electrophysiological and pharmacological profile. Pharmacological profiling distinguishes SLAI from SICI because only SLAI but not SICI decreases with blockade of cholinergic M1 receptors (Di Lazzaro et al., 2000). In addition, the classical benzodiazepine lorazepam increases SICI but decreases SLAI (Di Lazzaro et al., 2005a,b), and zolpidem, a positive modulator at the GABAAR with high affinity to the α1 subtype of the GABAAR, does not affect SICI but decreases SAI (Di Lazzaro et al., 2007). These data led to suppose the existence of at least partially distinct inhibitory neuronal circuits in the central nervous system. Recent electrophysiological findings strongly support the idea that SICI and SLAI are mediated through two distinct and reciprocally connected subtypes of GABA inhibitory interneurons with convergent projections onto the corticospinal neurons. Moreover, since SICI interneurons dominate on SLAI interneurons, it is suggested that SICI interneurons target the corticospinal neurons closer to their axon initial segment than the SLAI interneuron (Alle et al., 2009). Our findings are in line with these data, and support the idea that homotaurine elicits SLAI without interfering with SICI, thus acts on one of the distinct inhibitory circuits. In addition, it may be suggested a direct effect of homotaurine on cortical cholinergic terminals from basal forebrain neurons. The hypothesis seems unlikely since cholinergic neurons do not express receptors for GABA (Henderson, 1995; Mufson et al., 2003). We therefore suggest that homotaurine act directly on cortical interneurones through its high affinity binding to GABAARs, mimicking the effects of cholinergic transmission. Although homotaurine induced changes of intracortical inhibitory circuits, changes of LTP/LTD mechanisms were not observed. Earlier experiments performed in lab animals or in humans showed the potential of homotaurine to maintain LTP (Gervais et al., 2007), and also to interfere with Aβ fibril formation, two action that has been connected obeying to the amyloid cascade events (Aisen et al., 2007, 2011; Gauthier et al., 2009). Moreover, homotaurine produced measurable effects on cognition as well (Saumier et al., 2009). In our study, neuropsychological assessment showed no significant difference with respect to baseline. Reason of such discrepancies could be easily ascribed to the fact that available literature focused on AD patients, different doses of homotaurine has been used and for more prolonged time. All conditions that was supposed to interfere potentially with amyloid deposition. Here, we focused on MCI patients, for a relatively reduced time, conditions that highlighted the GABA-mediated effects of homotaurine rather than the potential anti-amyloid effects. Furthermore, it is noteworthy that we did not perform CSF biomarker analysis of our sample of patients. Recent studies on MCI patients, showed that in some cases pathologic Aβ 1–42 levels could be detected even 10–15 years before cognitive decline appears (Bondi et al., 2014). Thus, it may be supposed that our patients have a normal biomarker profile, and therefore the homotaurine efficacy resulted difficult to evaluate in both cognitive and electrophysiological tools. Particularly, the lack of effect on neuropsychological evaluation could be due to the fact that anti-amyloid activity contributes to stabilize a degenerative process while is not able to ameliorate measurable cognitive functions. On the other hand, it is conceivable to suppose that the traditional neuropsychological evaluation shows limits both in evaluating the cognitive impairment of MCI subjects, as recently discussed (Hansson et al., 2006) and their relief. However, more studies with larger sample MCI patients, using revised and adequate neuropsychological tools, possibly with different CSF biomarkers pattern (normal vs. pathologic Aβ 1–42 levels) could be of interest. Similarly, evaluation of homotaurine administration on SLAI in AD patients could help to better define the pharmacological homotaurine profile and also its indication in treatment of cognitive decline symptoms. In conclusion, this work represents an early pre-clinical study, and describes to our knowledge for the first time, the homotaurine effects on human inhibitory cortical activity, mimicking the effects observed for the cholinomimetic drugs (Di Lazzaro et al., 2002), useful to enhance cognition. Further clinical studies properly designed are needed however to fully evaluate homotaurine potential effects in treatment of MCI patients.

## Conflict of Interest Statement

The authors declare that the research was conducted in the absence of any commercial or financial relationships that could be construed as a potential conflict of interest.
